# Impact of Alcohol Intake and Drinking Patterns on Mortality From All Causes and Major Causes of Death in a Japanese Population

**DOI:** 10.2188/jea.JE20160200

**Published:** 2018-03-05

**Authors:** Eiko Saito, Manami Inoue, Norie Sawada, Hadrien Charvat, Taichi Shimazu, Taiki Yamaji, Motoki Iwasaki, Shizuka Sasazuki, Tetsuya Mizoue, Hiroyasu Iso, Shoichiro Tsugane

**Affiliations:** 1 Graduate School of Medicine, The University of Tokyo, Tokyo, Japan; 2 Epidemiology and Prevention Group, Center for Public Health Sciences, National Cancer Center, Tokyo, Japan; 3 Department of Epidemiology and Prevention, Center for Clinical Sciences, National Center for Global Health and Medicine, Tokyo, Japan; 4 Public Health, Department of Social and Environmental Medicine, Osaka University Graduate School of Medicine, Osaka, Japan

**Keywords:** alcohol, adult, mortality, cardiovascular diseases/mortality, follow-up studies, Japan/epidemiology, neoplasms/mortality, proportional hazards models

## Abstract

**Background:**

We examined the associations of alcohol consumption and liver holidays with all-cause mortality and with mortality due to cancer, heart disease, cerebrovascular disease, respiratory disease, and injury using a large-scale prospective study in Japan.

**Methods:**

We followed 102,849 Japanese who were aged between 40 and 69 years at baseline for 18.2 years on average, during which 15,203 deaths were reported. Associations between alcohol intake and mortality risk were assessed using a Cox proportional hazards model, with analysis by the number of liver holidays (in which a person abstains from drinking for several days a week).

**Results:**

A J-shaped association was observed between alcohol intake and total mortality in men (nondrinkers: reference; occasional drinkers: hazard ratio [HR] 0.74; 95% confidence interval [CI], 0.68–0.80; 1–149 g/week: HR 0.76; 95% CI, 0.71–0.81; 150–299 g/week: HR 0.75; 95% CI, 0.70–0.80; 300–449 g/week: HR 0.84; 95% CI, 0.78–0.91; 450–599 g/week: HR 0.92; 95% CI, 0.83–1.01; and ≥600 g/week: HR 1.19; 95% CI, 1.07–1.32) and in women (nondrinkers: reference; occasional: HR 0.75; 95% CI, 0.70–0.82; 1–149 g/week: HR 0.80; 95% CI, 0.73–0.88; 150–299 g/week: HR 0.91; 95% CI, 0.74–1.13; 300–449 g/week: HR 1.04; 95% CI, 0.73–1.48; and ≥450 g/week: HR 1.59; 95% CI, 1.07–2.38). In current drinkers, alcohol consumption was associated with a linear, positive increase in mortality risk from all causes, cancer, and cerebrovascular disease in both men and women, but not heart disease in men. Taking of liver holidays was associated with a lower risk of cancer and cerebrovascular disease mortality in men.

**Conclusions:**

Alcohol intake showed J-shaped associations with the risk of total mortality and three leading causes of death. However, heavy drinking increases the risk of mortality, which highlights the necessity of drinking in moderation coupled with liver holidays.

## INTRODUCTION

Many Asian countries have witnessed an increase in the level of alcohol consumption over the past decades, including Japan.^1^ Although alcohol is a major risk factor for cardiovascular diseases,^2^ cancer,^3^ and injury,^4^ dose-response analyses of alcohol consumption on mortality show varying results. While past studies have reported a reduced risk of total mortality,^5^^,^^6^ cardiovascular diseases,^7^ and cancer^8^ in light-to-moderate drinkers, heavy alcohol consumption has been positively associated with mortality from the same causes of death.^9^^,^^10^ This means that light-to-moderate drinkers may receive health benefits from alcohol intake, although the optimal range varies across studies and by population.

In assessing the impact of alcohol consumption on mortality outcomes, some questions need to be addressed in Asian populations. First, the impact of alcohol intake might be different in Asian than in Western populations. Asians have a high prevalence of people with facial flushing response due to inactive aldehyde dehydrogenase enzyme variants, which increases the blood level of acetaldehyde. Acetaldehyde is a major risk factor for cardiovascular and other diseases,^11^ and past studies pointed out the association of slow-metabolizing aldehyde dehydrogenase polymorphisms with myocardial infarction^12^ and site-specific cancers^13^^–^^15^ in Asians. However, the optimal limit to prevent premature mortality in Asian populations has not been well demonstrated. To date, only a few studies in Asia have assessed total and cause-specific mortality by alcohol consumption status, including one from the Japan Public Health Center-based Prospective Study.^5^^,^^16^^–^^22^ Even among existing studies, no study has comprehensively assessed the impact of alcohol intake on the five leading causes of death: cancer, heart disease, cerebrovascular disease, respiratory disease, and injury.^23^ Second, even among regular drinkers, the impact of alcohol consumption may differ by the number of drinking days in a week.^24^ Abstaining from drinking for several days a week, or the taking of so-called “liver holidays”, has been socially accepted, and is thus traditionally practiced in Japan to allow recuperation of the normal metabolic function of the liver. However, only one study, which employed JPHC study data, has reported the association of liver holidays with total mortality.^18^

Here, we aimed to estimate the impact of alcohol intake on total and five leading causes of death and to assess the association of liver holidays and risk of mortality using a large-scale, prospective cohort study in Japan.

## METHODS

### Study population

Details of the Japan Public Health Center-based Prospective Study have been described elsewhere.^25^^–^^27^ The baseline study for Cohort I started in 1990 and that for Cohort II started in 1993, covering a total of 140,420 participants (68,722 men and 71,698 women) in 11 public health center areas. The study enrolled participants aged 40 to 59 years in Cohort I and 40 to 69 years in Cohort II. Non-eligible participants were excluded (*n* = 291). Of the 140,129 eligible participants, 113,380 subjects (53,347 men and 60,033 women) completed the questionnaire. Of the subjects who returned the questionnaires, those who died, moved out of Japan, or lost to follow-up before the start of the follow-up period but who reported later were also excluded (*n* = 57). We excluded participants with self-reported past cancer, stroke, or myocardial infarction (*n* = 4,164). Subjects without information on alcohol consumption or intake of fruits, vegetables, total energy, meat, fish, and dairy products were also excluded (*n* = 6,310), leaving 102,849 participants for inclusion (48,309 men and 54,540 women). The study was approved by the Institutional Review Boards of the National Cancer Center in Tokyo and The University of Tokyo, Japan.

### Follow-up

Study participants were followed-up from enrollment in the baseline study (1990–1994) until the date of death or the end of follow-up (December 31, 2011), whichever came first. Subjects who migrated to other areas were followed through the residential registry. Of all subjects, 0.9% were lost to follow-up during the study period. Cause of death was ascertained using death certificates, with permission from the Ministry of Health, Labour and Welfare.^26^ The analysis included the five leading causes of death in Japan using the ICD10 classifications: cancer (C00–C97); heart disease (I20–I52); cerebrovascular disease (I60–I69); respiratory disease (J10–J18 and J40–J47), including pneumonia, influenza, chronic obstructive pulmonary disease, and associated conditions; and injuries and accidents (V01–X59, X60–X84, X85–Y09, Y85–Y86). Causes of death other than the above causes were also included.

### Assessment of exposure

The baseline questionnaire in Cohort I contained information on the frequency of alcohol intake: almost never, 1–3 days/month, 1–2 days/week, 3–4 days/week, 5–6 days/week, or every day. Subjects who drank more than 1–2 days/week were asked about the type of beverage and the average amount of intake per day. The questionnaire in Cohort II asked about the current drinking status, as never, former, or current drinkers. Former and current drinkers were then asked about the frequency of alcohol intake: 1–3 days/month, 1–2 days/week, 3–4 days/week, or almost every day, along with the type and amount of average consumption per day. In the 5-year and 10-year follow-up surveys for both cohorts, alcohol intake was assessed in accordance with the Cohort I baseline survey.

We defined the drinking status as follows: non-drinkers denote people who reported “almost never” in Cohort I and “never/stopped drinking” in Cohort II at baseline, or who reported “almost never” in 5-year and 10-year follow-up surveys; current drinkers denote people who reported drinking more than 1–3 days/month at the time of the survey. In the subgroup analysis, former drinkers were defined as people who stopped drinking before baseline in Cohort II. To calibrate alcohol intake, we first assigned a score for each category of intake frequency: 0 for almost never, 0.5 for 1–3 days/month, 1.5 for 1–2 days/week, 3.5 for 3–4 days/week, 5.5 for 5–6 days/week, and 7.0 for every day in the Cohort I baseline; and 0 for almost never, 0.5 for 1–3 days/month, 1.5 for 1–2 days/week, 3.5 for 3–4 days/week, and 6.0 for almost every day in the Cohort II baseline. For 5-year and 10-year follow-up surveys, we assigned the same scores as in the Cohort I baseline survey. Second, for regular drinkers who drank more than once a week, alcohol intake was estimated by multiplying the grams of ethanol contained in each type of drink. In the JPHC study, one drink is assumed to contain 23 g ethanol for 180 mL (one *gou*) of *sake*, 36 g ethanol for 180 mL of *shochu* and *awamori*, 10 g ethanol for 30 mL of whisky or brandy, 6 g ethanol for 60 mL of wine, or 23 g ethanol for 633 mL of beer (a large bottle). Third, we estimated the weekly ethanol intake at each survey year by multiplying the quantity by score. Fourth, cumulative average intake of alcohol was estimated by taking the average of the available time points starting from the baseline survey. For instance, cumulative average alcohol intake at the time of 5-year follow-up was calculated by averaging alcohol intake at baseline and 5 years, and the same intake at 10-year follow-up onwards was calculated by averaging the intake at baseline, and 5-year and 10-year follow-ups, or any combination of available time points, and used as a time-dependent variable.^28^ Subjects were classified for cumulative average intake of alcohol into seven groups for men: non-drinkers, occasional drinkers (1–3 day/month), and five groups of regular drinkers (1–149 g/week ethanol, 150–299 g/week, 300–449 g/week, 450–599 g/week, and 600 g/week or more). Cumulative average intake was categorized into six groups for women: non-drinkers, occasional drinkers (1–3 day/month), and four groups of regular drinkers (1–149 g/week, 150–299 g/week, 300–449 g/week, and 450 g/week or more). An ethanol intake of 150 g/week is equivalent to having less than one bottle of beer or one *gou* of *sake* per day, 300 g/week to two bottles of beer or two *gou* of *sake* per day, and 450 g/week to three bottles of beer or three *gou* of *sake* per day.

Further, a drinking pattern was measured from the cumulative average intake of alcohol and the cumulative average number of ‘liver holidays’, defined as the number of days without drinking alcohol, per week (no holiday, 1–2 days per week, 3–4 days per week, and 5–6 days per week) among regular drinkers who consume alcohol more than once a week. We conducted a stratified analysis by light-drinking men (<150 g/week), moderate-drinking men (150–299 g/week), and heavy-drinking men (300+ g/week), while analysis of drinking patterns in women included those with all amount categories to allow a sufficient number of cases for analysis.

Dietary records for 28 days (repeating 1-week dietary records at 3-month intervals) or 14-day dietary records were used to validate the baseline, 5-year, and 10-year questionnaires. Spearman rank correlation coefficients of alcohol intake between the questionnaires and dietary records were 0.79 for men and 0.44 for women in Cohort I^29^ and 0.59 for men and 0.40 in women in Cohort II,^30^ both for the baseline survey. For the 5-year follow-up survey, the correlation coefficients of alcohol intake were 0.77 for men and 0.51 for women.^31^ The reproducibility of alcohol intake in Cohort I was 0.66 between 1990 and 1995 at a 5-year interval, and 0.63 in Cohort II between 1993 and 1997 at a 4-year interval.^29^ The reproducibility of the comprehensive food frequency questionnaires for the 5-year follow-up survey, administered at a 1-year interval, was 0.79 in men and 0.71 in women.^32^

### Statistical analysis

Associations between cumulative average alcohol intake, drinking patterns, and the risk of mortality were measured from hazard ratios (HRs) and 95% confidence intervals (CIs) using a Cox proportional hazards regression model. Tests for non-linearity were conducted by assigning the scores for each category of cumulative alcohol intake from zero for never drinkers to 5 for the highest intake category, and then alcohol intake was used as a continuous variable; the likelihood ratio test was used to compare the model with only the linear term and the model with both the linear and quadratic terms. Tests for linear trend in drinkers were conducted using the same scores but restricting the subjects only to current drinkers. For the liver holidays, we tested for linear trends by assigning the scores for each category of the number of liver holidays taken, from zero for no liver holidays to three for 5–6 liver holidays per week. The model was adjusted for the following potential confounders: age at baseline (continuous); public health center; smoking status (never, former, <20 cigarettes per day, and ≥20 cigarettes per day); BMI (in kg/m^2^; <18.5, 18.5 to <25, 25 to <30, and ≥30); flushing response after drinking (no or yes); history of hypertension (no or yes); history of diabetes (no or yes); leisure-time sports (<almost daily or almost daily); consumption of green tea and coffee (almost never, ≥1 cup/week, and ≥1 cup/day); total energy consumption per day (continuous); log-transformed, daily consumption of fruit, vegetables, meat, fish, and dairy products, with adjustment of total energy intake using the residual method (continuous); and job status at baseline (employed or unemployed). The same analysis was conducted after excluding deaths occurring within 5 years after baseline, to reduce the chance of reverse causality from ongoing but subclinical illnesses. Further, we conducted a secondary analysis excluding past drinkers in Cohort II, and further analyses by smoking status (current smokers and never smokers in men, and never smokers in women). We estimated *P* for interaction by using likelihood-ratio tests which compared the models with and without cross-product terms for smoking status, with alcohol intake as a continuous term. Tests for non-proportional hazards by Therneau and Grambsch were used to evaluate departures from proportional hazards assumption, and no violation of the assumption was observed. Since the questionnaires for both cohorts were designed differently, we evaluated whether the associations varied between cohorts by combining the cohort-specific estimates in a fixed-effects meta-analysis and then performing Chi-square tests for heterogeneity. Cohort-specific HRs for alcohol intake and all-cause mortality were weighted by the inverse of the sum of their variance. For sub-analyses by smoking status, without abstainers during follow-up, and for tests for heterogeneity, we grouped men who drink ≥450 g/week into a single category to allow enough number of cases. All *P*-values were two-sided, with values smaller than 0.05 indicating statistical significance. All analyses were conducted with STATA version 14.0 software (StataCorp LP, College Station, TX, USA).

## RESULTS

Table 1 summarizes the characteristics of study participants by alcohol consumption status. Participants with larger alcohol intake were younger, smoked more, and reported a higher prevalence of hypertension for both men and women. During the follow-up period (18.2 years on average; total person-years: 1,867,366), a total of 15,203 deaths were reported. Of these, 6,228 deaths were reported due to cancer, 1,899 to heart disease, 1,493 to cerebrovascular disease, 948 to respiratory disease, 1,141 to injury, and 3,494 to other causes. Of all the participants who completed the baseline questionnaire, 80.7% returned the 5-year follow-up questionnaire and 76.9% returned the 10-year follow-up questionnaire.

**Table 1.** Baseline characteristics of participants by alcohol consumption status

Characteristic	Cumulative Average Intake							

	Non-drinkers	Occasional drinkers	0–149 g/week	150–299 g/week	300–449 g/week	450–599 g/week	≥600 g/week	*P*-value^a^
**Men (*n* = 48,300)**	**6,492**	**5,010**	**11,727**	**12,171**	**7,747**	**3,041**	**2,112**	
Alcohol consumption per week, median	0.0	2.9	77.8	218.5	368.0	504.0	698.0	<0.001
Age, years, mean	52.6	51.5	51.1	51.2	50.4	49.8	49.9	<0.001
Current smoker, %	50.2	47.4	44.3	54.1	60.8	61.5	61.8	<0.001
Body mass index, kg/m^2^, mean	23.3	23.7	23.4	23.4	23.5	23.6	23.9	<0.001
Flushing response to alcohol, %	73.3	70.0	56.9	44.0	37.8	35.2	31.1	<0.001
History of hypertension, %	12.3	12.1	14.7	18.5	20.4	19.6	18.8	<0.001
History of diabetes, %	6.6	6.3	6.0	5.7	5.4	6.6	8.4	0.001
Sports or physical exercise almost daily, %	5.4	4.9	4.9	4.5	4.9	4.4	4.3	0.056
Coffee >1 time/day, %	45.6	44.2	44.8	39.9	36.5	37.4	36.5	<0.001
Green tea >1 time/day, %	73.1	72.3	73.7	74.5	72.5	70.0	64.3	<0.001
Dietary intake^b^								
Total energy intake, kcal/d, mean	1,725	1,739	1,801	1,953	2,078	2,180	2,270	<0.001
Fruits, g/d, mean	76.1	72.9	71.4	63.4	59.5	55.0	51.9	<0.001
Vegetables, g/d, mean	74.1	76.6	75.0	75.1	74.3	68.2	60.0	<0.001
Meat, g/d, mean	30.0	31.2	29.8	28.2	27.1	25.6	25.0	<0.001
Fish, g/d, mean	66.1	63.8	68.7	69.1	69.8	67.5	66.1	<0.001
Dairy products, g/d, mean	116.5	123.0	115.4	93.0	74.9	65.2	58.7	<0.001
Employed at the time of baseline, %	86.8	91.4	93.4	94.3	95.3	95.6	93.0	<0.001
**Women (*n* = 54,540)**	**33,723**	**10,387**	**8,696**	**1,205**	**357**	**172**		
Alcohol consumption per week, median	0.0	0.0	31.8	189.5	354.0	551.0		<0.001
Age, years, mean	52.6	50.6	48.7	47.6	47.5	47.3		<0.001
Current smoker, %	4.5	6.3	11.9	32.5	48.7	49.4		<0.001
Body mass index, kg/m^2^, mean	23.5	23.5	22.8	22.8	23.0	23.2		<0.001
Flushing response to alcohol, %	37.7	39.5	34.8	29.0	34.2	31.4		<0.001
History of hypertension, %	16.2	13.6	11.4	13.5	14.9	19.2		<0.001
History of diabetes, %	3.1	2.5	1.7	1.9	3.6	2.3		<0.001
Sports or physical exercise almost daily, %	4.7	4.6	4.0	3.8	3.6	5.2		0.001
Coffee >1 time/day, %	36.0	41.0	52.5	55.1	47.3	39.0		<0.001
Green tea >1 time/day, %	74.6	75.4	75.7	65.8	56.0	56.4		<0.001
Dietary intake^b^								
Total energy intake, kcal/d, mean	1,215	1,251	1,274	1,338	1,455	1,486		<0.001
Fruits, g/d, mean	138.9	145.6	136.2	112.8	97.5	82.8		<0.001
Vegetables, g/d, mean	100.9	107.6	105.8	98.9	93.8	82.0		<0.001
Meat, g/d, mean	29.4	30.6	30.5	28.9	24.6	24.0		<0.001
Fish, g/d, mean	61.6	61.2	61.6	58.5	56.7	52.3		<0.001
Dairy products, g/d, mean	297.6	309.1	309.6	229.3	188.1	162.5		<0.001
Employed at the time of baseline, %	55.1	61.8	63.4	67.0	74.0	72.1		<0.001

^a^ANOVA or chi-square-test.

^b^All mean total intakes of food are energy adjusted.

HRs with 95% CIs for the association between cumulative average intake of alcohol and all-cause and cause-specific mortality are presented in Table 2 (men) and Table 3 (women). A J-shaped association was observed between cumulative average alcohol intake and total mortality in both men (non-drinkers: reference; occasional: HR 0.74; 95% CI, 0.68–0.80; 1–149 g/week: HR 0.76; 95% CI, 0.71–0.81; 150–299 g/week: HR 0.75; 95% CI, 0.70–0.80; 300–449 g/week: HR 0.84; 95% CI, 0.78–0.91; 450–599 g/week: HR 0.92; 95% CI, 0.83–1.01; and ≥600 g/week: HR 1.19; 95% CI, 1.07–1.32) and in women (non-drinkers: reference; occasional: HR 0.75; 95% CI, 0.70–0.82; 1–149 g/week: HR 0.80; 95% CI, 0.73–0.88; 150–299 g/week: HR 0.91; 95% CI, 0.74–1.13; 300–449 g/week: HR 1.04; 95% CI, 0.73–1.48; and ≥450 g/week: HR 1.59; 95% CI, 1.07–2.38), after adjustment for confounders. These associations were consistent even after excluding deaths occurring within 5 years of baseline in both men (non-drinkers: reference; occasional: HR 0.70; 95% CI, 0.64–0.76; 1–149 g/week: HR 0.78; 95% CI, 0.73–0.83; 150–299 g/week: HR 0.73; 95% CI, 0.68–0.78; 300–449 g/week: HR 0.77; 95% CI, 0.71–0.83; 450–599 g/week: HR 1.01; 95% CI, 0.92–1.12; and ≥600 g/week: HR 1.07; 95% CI, 0.96–1.20) and in women (non-drinkers: reference; occasional: HR 0.59; 95% CI, 0.53–0.64; 1–149 g/week: HR 0.72; 95% CI, 0.65–0.80; 150–299 g/week: HR 0.91; 95% CI, 0.73–1.14; 300–449 g/week: HR 1.00; 95% CI, 0.69–1.45; and ≥450 g/week: HR 1.19; 95% CI, 0.75–1.89). We found no evidence of heterogeneity between Cohort I and Cohort II on the association between alcohol intake and total mortality (*P*-value = 0.963).

**Table 2.** Adjusted hazard ratios of mortality by alcohol consumption status (men)

	Cumulative Average Intake														

	Non-drinkers	Occasional drinkers		0–149 g/week		150–299 g/week		300–449 g/week		450–599 g/week		≥600 g/week		*P* for non-linear trend	*P* for linear trend in drinkers^e^
	HR^a^	HR	95% CI	HR	95% CI	HR	95% CI	HR	95% CI	HR	95% CI	HR	95% CI		
All-cause mortality															
Person-years (*n* = 855,250)	109,253	90,714		208,202		218,290		138,842		54,067		35,729			
Number of cases (*n* = 9,768)	1,735	918		2,085		2,298		1,550		637		545			
Model 1 adjusted HRs^b^	1.00	0.71	(0.66–0.77)	0.73	(0.69–0.78)	0.78	(0.74–0.84)	0.92	(0.86–0.99)	1.03	(0.94–1.13)	1.37	(1.24–1.51)	<0.001	<0.001
Model 2 adjusted HRs^c^	1.00	0.74	(0.68–0.80)	0.76	(0.71–0.81)	0.75	(0.70–0.80)	0.84	(0.78–0.91)	0.92	(0.83–1.01)	1.19	(1.07–1.32)	<0.001	<0.001
Model 2 adjusted HRs^d^	1.00	0.70	(0.64–0.76)	0.78	(0.73–0.83)	0.73	(0.68–0.78)	0.77	(0.71–0.83)	1.01	(0.92–1.12)	1.07	(0.96–1.20)	<0.001	<0.001
Cancer															
Number of cases (*n* = 4,054)	684	320		905		991		677		265		212			
Model 1 adjusted HRs^b^	1.00	0.65	(0.57–0.74)	0.81	(0.74–0.89)	0.84	(0.76–0.92)	0.97	(0.87–1.08)	1.16	(1.01–1.33)	1.29	(1.10–1.51)	<0.001	<0.001
Model 2 adjusted HRs^c^	1.00	0.67	(0.59–0.77)	0.86	(0.78–0.95)	0.82	(0.74–0.91)	0.91	(0.81–1.02)	1.06	(0.91–1.22)	1.17	(0.99–1.38)	<0.001	<0.001
Model 2 adjusted HRs^d^	1.00	0.68	(0.59–0.79)	0.92	(0.82–1.02)	0.84	(0.75–0.94)	0.89	(0.78–1.01)	1.19	(1.02–1.38)	1.12	(0.94–1.35)	<0.001	<0.001
Heart disease															
Number of cases (*n* = 1,203)	224	131		247		267		193		80		61			
Model 1 adjusted HRs^b^	1.00	0.72	(0.58–0.89)	0.65	(0.55–0.78)	0.64	(0.54–0.76)	0.80	(0.66–0.97)	0.85	(0.66–1.11)	1.14	(0.87–1.51)	<0.001	<0.001
Model 2 adjusted HRs^c^	1.00	0.73	(0.59–0.91)	0.67	(0.56–0.80)	0.59	(0.49–0.71)	0.71	(0.57–0.87)	0.72	(0.55–0.95)	0.93	(0.69–1.24)	<0.001	0.112
Model 2 adjusted HRs^d^	1.00	0.71	(0.56–0.91)	0.69	(0.57–0.83)	0.61	(0.50–0.74)	0.74	(0.60–0.93)	0.77	(0.58–1.03)	0.87	(0.63–1.21)	<0.001	0.104
Cerebrovascular disease															
Number of cases (*n* = 905)	151	78		181		225		146		59		65			
Model 1 adjusted HRs^b^	1.00	0.61	(0.46–0.80)	0.75	(0.61–0.93)	0.87	(0.71–1.06)	0.96	(0.77–1.21)	1.07	(0.80–1.44)	1.70	(1.26–2.30)	<0.001	<0.001
Model 2 adjusted HRs^c^	1.00	0.61	(0.46–0.82)	0.74	(0.60–0.92)	0.78	(0.63–0.96)	0.83	(0.65–1.06)	0.90	(0.65–1.23)	1.35	(0.98–1.87)	<0.001	<0.001
Model 2 adjusted HRs^d^	1.00	0.60	(0.44–0.82)	0.75	(0.59–0.94)	0.79	(0.62–0.99)	0.75	(0.57–0.98)	0.97	(0.69–1.35)	1.36	(0.95–1.93)	<0.001	0.002
Respiratory disease															
Number of cases (*n* = 672)	143	66		159		151		93		31		29			
Model 1 adjusted HRs^b^	1.00	0.49	(0.36–0.67)	0.66	(0.53–0.81)	0.59	(0.47–0.74)	0.57	(0.43–0.75)	0.75	(0.53–1.07)	0.82	(0.55–1.22)	<0.001	0.115
Model 2 adjusted HRs^c^	1.00	0.53	(0.38–0.72)	0.65	(0.52–0.81)	0.54	(0.43–0.69)	0.51	(0.38–0.68)	0.67	(0.46–0.97)	0.72	(0.47–1.10)	<0.001	0.645
Model 2 adjusted HRs^d^	1.00	0.55	(0.39–0.75)	0.68	(0.54–0.86)	0.55	(0.43–0.70)	0.48	(0.35–0.66)	0.65	(0.44–0.96)	0.63	(0.39–1.00)	<0.001	0.641
Injury															
Number of cases (*n* = 805)	132	73		158		180		145		68		49			
Model 1 adjusted HRs^b^	1.00	0.81	(0.61–1.07)	0.73	(0.58–0.92)	0.75	(0.60–0.94)	0.99	(0.78–1.26)	1.06	(0.78–1.44)	1.46	(1.06–2.02)	<0.001	<0.001
Model 2 adjusted HRs^c^	1.00	0.83	(0.63–1.10)	0.75	(0.60–0.95)	0.73	(0.58–0.93)	0.92	(0.72–1.19)	0.97	(0.70–1.33)	1.26	(0.89–1.78)	<0.001	0.012
Model 2 adjusted HRs^d^	1.00	0.92	(0.67–1.26)	0.78	(0.60–1.02)	0.79	(0.60–1.03)	0.97	(0.72–1.29)	1.22	(0.86–1.73)	1.26	(0.84–1.89)	0.001	0.013
Other causes															
Number of cases (*n* = 2,130)	401	250		435		484		297		134		129			
Model 1 adjusted HRs^b^	1.00	0.70	(0.60–0.83)	0.61	(0.54–0.70)	0.65	(0.57–0.74)	0.69	(0.59–0.80)	0.93	(0.77–1.12)	1.28	(1.05–1.56)	<0.001	<0.001
Model 2 adjusted HRs^c^	1.00	0.73	(0.62–0.86)	0.62	(0.54–0.70)	0.60	(0.52–0.68)	0.62	(0.53–0.73)	0.81	(0.66–0.99)	1.07	(0.86–1.32)	<0.001	0.002
Model 2 adjusted HRs^d^	1.00	0.68	(0.57–0.81)	0.60	(0.52–0.69)	0.60	(0.51–0.69)	0.61	(0.52–0.72)	0.85	(0.69–1.04)	0.98	(0.78–1.23)	<0.001	0.001

CI, confidence interval; HR, hazard ratio.

^a^Cox proportional hazards models were used. Intake categories are presented by cumulative alcohol consumption updated to 10-year follow-up survey or available time points.

^b^Model 1: adjusted for age (years, continuous) and public health center area.

^c^Model 2: adjusted for smoking status (never, former, <20 cigarettes/day, ≥20 cigarettes/day), BMI (<18.5, 18.5–<25, 25–<30, 30+), history of hypertension, flushing response, history of diabetes, leisure-time sports or physical exercise (<almost daily, almost daily), intake of coffee and green tea (almost never, ≥1 cup/wk, and ≥1 cup/d), energy intake (continuous), intakes of fruits, vegetables, fish, meat, dairy products (continuous), and job status (employed or unemployed) in addition to the adjustment factors in Model 1.

^d^Model 2 excluding deaths within 5 years of baseline.

^e^*P* for linear trend in drinkers was assessed only among current drinkers (non-drinkers were excluded from the analysis).

**Table 3.** Adjusted hazard ratios of mortality by alcohol consumption status (women)

	Cumulative Average Intake												

	Non-drinkers	Occasional drinkers		1–149 g/week		150–299 g/week		300–449 g/week		≥450 g/week		*P* for non-linear trend	*P* for linear trend in drinkers^e^
	HR^a^	HR	95% CI	HR	95% CI	HR	95% CI	HR	95% CI	HR	95% CI		
All-cause mortality													
Person-years (*n* = 1,012,269)	621,932	197,560		161,603		21,805		6,332		3,037			
Number of cases (*n* = 5,434)	3,985	755		547		90		32		25			
Model 1 adjusted HRs^b^	1.00	0.75	(0.69–0.81)	0.82	(0.75–0.90)	1.17	(0.95–1.44)	1.50	(1.06–2.13)	2.49	(1.68–3.69)	<0.001	0.006
Model 2 adjusted HRs^c^	1.00	0.75	(0.70–0.82)	0.80	(0.73–0.88)	0.91	(0.74–1.13)	1.04	(0.73–1.48)	1.59	(1.07–2.38)	<0.001	<0.001
Model 2 adjusted HRs^d^	1.00	0.59	(0.53–0.64)	0.72	(0.65–0.80)	0.91	(0.73–1.14)	1.00	(0.69–1.45)	1.19	(0.75–1.89)	<0.001	<0.001
Cancer													
Number of cases (*n* = 2,174)	1,539	334		242		34		16		9			
Model 1 adjusted HRs^b^	1.00	0.68	(0.59–0.78)	0.74	(0.64–0.86)	1.03	(0.73–1.44)	1.62	(0.99–2.65)	1.54	(0.77–3.09)	<0.001	0.001
Model 2 adjusted HRs^c^	1.00	0.67	(0.59–0.77)	0.71	(0.61–0.83)	0.87	(0.62–1.23)	1.25	(0.76–2.08)	1.17	(0.57–2.37)	<0.001	<0.001
Model 2 adjusted HRs^d^	1.00	0.66	(0.57–0.77)	0.73	(0.62–0.85)	0.90	(0.62–1.30)	1.28	(0.74–2.21)	1.02	(0.45–2.32)	<0.001	<0.001
Heart disease													
Number of cases (*n* = 696)	525	94		62		8		3		4			
Model 1 adjusted HRs^b^	1.00	0.56	(0.43–0.74)	0.70	(0.52–0.94)	0.98	(0.49–1.98)	1.35	(0.43–4.22)	3.36	(1.25–9.01)	<0.001	0.022
Model 2 adjusted HRs^c^	1.00	0.58	(0.44–0.76)	0.68	(0.51–0.92)	0.71	(0.35–1.44)	0.84	(0.27–2.67)	2.05	(0.74–5.62)	<0.001	0.004
Model 2 adjusted HRs^d^	1.00	0.58	(0.43–0.76)	0.70	(0.52–0.96)	0.80	(0.39–1.65)	0.96	(0.30–3.05)	1.80	(0.56–5.76)	0.001	0.009
Cerebrovascular disease													
Number of cases (*n* = 588)	441	75		45		17		4		6			
Model 1 adjusted HRs^b^	1.00	0.48	(0.36–0.66)	0.61	(0.44–0.85)	2.10	(1.27–3.48)	1.41	(0.45–4.39)	5.57	(2.48–12.51)	<0.001	0.287
Model 2 adjusted HRs^c^	1.00	0.49	(0.36–0.66)	0.56	(0.40–0.78)	1.34	(0.79–2.27)	0.81	(0.25–2.58)	2.70	(1.15–6.30)	<0.001	0.016
Model 2 adjusted HRs^d^	1.00	0.47	(0.34–0.64)	0.54	(0.38–0.77)	1.43	(0.83–2.47)	0.62	(0.15–2.55)	3.10	(1.32–7.32)	<0.001	0.020
Respiratory disease													
Number of cases (*n* = 276)	238	25		10		3		0		0			
Model 1 adjusted HRs^b^	1.00	0.36	(0.21–0.59)	0.34	(0.18–0.65)	0.95	(0.30–2.98)	n/a		n/a		0.057	<0.001
Model 2 adjusted HRs^c^	1.00	0.37	(0.22–0.62)	0.34	(0.18–0.65)	0.75	(0.23–2.41)	n/a		n/a		0.111	<0.001
Model 2 adjusted HRs^d^	1.00	0.40	(0.24–0.66)	0.33	(0.17–0.66)	0.81	(0.25–2.62)	n/a		n/a		0.315	<0.001
Injury													
Number of cases (*n* = 336)	225	53		45		8		4		1			
Model 1 adjusted HRs^b^	1.00	0.67	(0.47–0.95)	0.95	(0.68–1.34)	1.58	(0.78–3.22)	2.71	(1.00–7.32)	1.39	(0.19–9.91)	0.018	0.704
Model 2 adjusted HRs^c^	1.00	0.66	(0.47–0.94)	0.84	(0.59–1.19)	0.96	(0.46–2.01)	1.40	(0.50–3.94)	0.66	(0.09–4.87)	0.128	0.274
Model 2 adjusted HRs^d^	1.00	0.64	(0.44–0.94)	0.80	(0.54–1.17)	0.80	(0.34–1.87)	0.78	(0.19–3.30)	n/a		0.604	0.056
Other causes													
Number of cases (*n* = 1,364)	1,017	174		143		20		5		5			
Model 1 adjusted HRs^b^	1.00	0.47	(0.39–0.58)	0.80	(0.66–0.97)	0.88	(0.54–1.45)	1.59	(0.79–3.19)	1.50	(0.56–4.01)	<0.001	<0.001
Model 2 adjusted HRs^c^	1.00	0.49	(0.40–0.60)	0.81	(0.67–0.98)	0.71	(0.43–1.18)	1.10	(0.54–2.24)	0.98	(0.36–2.66)	<0.001	<0.001
Model 2 adjusted HRs^d^	1.00	0.51	(0.41–0.62)	0.85	(0.70–1.04)	0.73	(0.43–1.23)	1.19	(0.58–2.43)	1.05	(0.39–2.84)	<0.001	0.001

CI, confidence interval; HR, hazard ratio.

^a^Cox proportional hazards models were used. Intake categories are presented by cumulative alcohol consumption updated to 10-year follow-up survey or available time points.

^b^Model 1: adjusted for age (years, continuous) and public health center area.

^c^Model 2: adjusted for smoking status (never, former, <20 cigarettes/day, ≥20 cigarettes/day), BMI (<18.5, 18.5–<25, 25–<30, 30+), history of hypertension, flushing response, history of diabetes, leisure-time sports or physical exercise (<almost daily, almost daily), intake of coffee and green tea (almost never, ≥1 cup/wk, and ≥1 cup/d), energy intake (continuous), intakes of fruits, vegetables, fish, meat, dairy products (continuous), and job status (employed or unemployed) in addition to the adjustment factors in Model 1.

^d^Model 2 excluding deaths within 5 years of baseline.

^e^*P* for linear trend in drinkers was assessed only among current drinkers (non-drinkers were excluded from the analysis).

Similarly, the multivariate model showed that the cumulative average consumption of alcohol had a J-shaped association with mortality from cancer and cerebrovascular disease in men, with the risks lower in occasional drinkers and those who drank 1–149 g/week to 150–299 g/week compared to non-drinkers, and increase in mortality risk with ≥450 g/week for cancer and ≥600 g/week for cerebrovascular disease. On the other hand, a U-shaped association was seen in mortality from heart disease and respiratory disease in men. The adjusted HRs in women showed the same J-shaped association with mortality from all causes, cancer, heart disease, and cerebrovascular disease, in which the risk reduction remained in women who drank 1–149 g/week compared to non-drinkers. When we restricted our analysis only to current drinkers, tests for linear trend showed a linear increase in the risk of mortality due to all causes, cancer, cerebrovascular disease, and injury in men, and to all causes, cancer, cerebrovascular disease, heart disease, and respiratory disease in women. The analysis of drinking patterns showed that and having 5–6 days of liver holiday a week was associated with a lower risk of cancer and cerebrovascular disease mortality in light-drinking men, while having 1–2 days of liver holiday a week was associated with a lower risk of total mortality in light-drinking men and a lower risk of cancer and cerebrovascular disease mortality regardless of the weekly amount intake (Table 4).

**Table 4.** Adjusted hazard ratios by the number of liver holidays per week in regular drinkers

	Number of liver holidays							

	No holiday	1–2 days/wk		3–4 days/wk		5–6 days/wk		*P* for linear trend
	HR^a^	HR	95% CI	HR	95% CI	HR	95% CI	
**Men, light drinkers (<150 g/week)**								
All-cause, number of cases (*n* = 2,085)	218	464		738		665		
Multivariate HRs^b^	1.00	0.77	(0.65–0.91)	0.95	(0.81–1.11)	0.93	(0.79–1.09)	0.272
Cancer, number of cases (*n* = 905)	99	208		320		278		
Multivariate HRs^b^	1.00	0.71	(0.57–0.88)	0.83	(0.69–1.02)	0.72	(0.58–0.88)	0.031
Heart disease, number of cases (*n* = 247)	28	43		89		87		
Multivariate HRs^b^	1.00	0.76	(0.48–1.18)	0.88	(0.59–1.32)	1.05	(0.69–1.58)	0.402
Cerebrovascular disease, number of cases (*n* = 181)	20	47		66		48		
Multivariate HRs^b^	1.00	0.60	(0.38–0.97)	0.68	(0.45–1.03)	0.61	(0.38–0.96)	0.094
**Men, moderate drinkers (150–299 g/week)**								
All-cause, number of cases (*n* = 2,298)	645	1044		518		91		
Multivariate HRs^b^	1.00	0.96	(0.86–1.07)	1.26	(1.11–1.43)	1.14	(0.91–1.44)	0.001
Cancer, number of cases (*n* = 991)	307	433		212		39		
Multivariate HRs^b^	1.00	0.74	(0.63–0.86)	0.95	(0.79–1.14)	0.71	(0.46–1.08)	0.101
Heart disease, number of cases (*n* = 267)	69	128		58		12		
Multivariate HRs^b^	1.00	0.86	(0.64–1.16)	0.98	(0.69–1.41)	0.83	(0.37–1.85)	0.707
Cerebrovascular disease, number of cases (*n* = 225)	68	96		55		6		
Multivariate HRs^b^	1.00	0.60	(0.43–0.84)	1.03	(0.72–1.47)	0.35	(0.11–1.12)	0.299
**Men, heavy drinkers (≥300 g/week)**								
All-cause, number of cases (*n* = 2,732)	1,140	1,300		264		28		
Multivariate HRs^b^	1.00	0.98	(0.89–1.07)	1.17	(1.02–1.36)	1.00	(0.68–1.47)	0.205
Cancer, number of cases (*n* = 1,154)	490	547		101		16		
Multivariate HRs^b^	1.00	0.75	(0.65–0.87)	0.95	(0.76–1.20)	1.42	(0.79–2.53)	0.061
Heart disease, number of cases (*n* = 334)	136	160		36		2		
Multivariate HRs^b^	1.00	0.79	(0.61–1.02)	1.01	(0.67–1.53)	1.25	(0.46–3.43)	0.525
Cerebrovascular disease, number of cases (*n* = 270)	126	117		25		2		
Multivariate HRs^b^	1.00	0.67	(0.50–0.90)	1.02	(0.65–1.61)	0.41	(0.06–2.97)	0.135
**Women**								
All-cause, number of cases (*n* = 694)	50	155		237		252		
Multivariate HRs^b^	1.00	1.12	(0.80–1.55)	1.15	(0.83–1.60)	1.09	(0.78–1.53)	0.850
Cancer, number of cases (*n* = 301)	24	67		100		110		
Multivariate HRs^b^	1.00	0.88	(0.58–1.36)	0.75	(0.49–1.16)	0.75	(0.48–1.17)	0.183
Heart disease, number of cases (*n* = 77)	2	19		26		30		
Multivariate HRs^b^	1.00	2.85	(0.83–9.76)	1.83	(0.52–6.42)	1.53	(0.42–5.55)	0.498
Cerebrovascular disease, number of cases (*n* = 72)	11	14		22		25		
Multivariate HRs^b^	1.00	0.51	(0.22–1.15)	0.73	(0.33–1.61)	0.70	(0.30–1.65)	0.744

CI, confidence interval; HR, hazard ratio.

^a^Cox proportional hazards models were used. Intake categories are presented by cumulative alcohol consumption updated to 10-year follow-up survey or available time points.

^b^Adjusted for age (years, continuous), public health center area, smoking status (never, former, <20 cigarettes/day, ≥20 cigarettes/day), BMI (<18.5, 18.5–<25, 25–<30, 30+), cumulative average alcohol intake (1–149 g/w, 150–299 g/w, 300–449 g/w, 450–599 g/w, 600+ g/w), flushing response, history of hypertension, history of diabetes, leisure-time sports or physical exercise (<almost daily, almost daily), intake of coffee and green tea (almost never, ≥1 cup/w, and ≥1 cup/d), energy intake (continuous), intakes of fruits, vegetables, fish, meat, dairy products (continuous), and job status (employed or unemployed) in addition to the adjustment factors in Model 1.

In a subgroup analysis excluding past drinkers in Cohort II (eTable 1 and eTable 2), the same associations were observed in all-cause and cancer, heart disease, cerebrovascular disease, and respiratory disease mortality in men. In women, the J-shaped associations with alcohol intake and mortality were consistent even after excluding past drinkers. The J-shaped associations with total mortality remained the same regardless of smoking status in both men and women (eTable 3). For those who used to drink at the time of baseline but abstained during follow-up, the same J-shaped associations were observed for both men and women.

## DISCUSSION

This is the first study in Asia to investigate the impact of alcohol intake on mortality from five leading causes of death, with measurements of intake over 10 years during the follow-up period. Our results from 102,849 Japanese men and women aged between 40 and 69 years showed a J-shaped association between alcohol intake and mortality from all causes, cancer, and cerebrovascular disease and a U-shaped association with heart disease and respiratory mortality in men. We also reported a J-shaped association with mortality from all causes, cancer, heart disease, and cerebrovascular disease in women, which corroborates previous reports.^5^^,^^10^

The optimal limit of alcohol intake in women (up to ∼150 g/week) is consistent with that in Western populations: a large-scale cohort study in Sweden showed no significant rise in total mortality risk among those who drank up to ∼140 g per week,^33^ and a meta-analysis of nine prospective cohort studies in the United States and Europe with repeated measures reported that alcohol consumption up to ∼200 g per week was associated with a lower risk of total mortality.^34^ However, we observed that the mortality risk becomes elevated with more than 450 g per week of alcohol intake in men relative to non-drinkers, which is consistent with the results obtained from prospective cohort studies in Japan.^5^^,^^17^^,^^18^ The J-shaped associations in total mortality might be confounded by smoking status, since heavy drinkers tend to smoke more. However, our stratified analysis by smoking status consistently showed the same associations.

Caution should be raised, though, that such J-shaped associations could have occurred because non-drinkers contain a high-risk group of former drinkers who quit drinking due to ill health.^35^ However, although the number was limited, 14.6% of men and 8.3% of women in Cohort II who abstained before baseline died during follow-up, which is lower than the mortality witnessed in the overall study participants (20.2% in men and 17.9% in women). Our analysis excluding abstainers before and during the follow-up period also showed similar associations.

Relatively high tolerance for alcohol in Japanese men suggests a paradox: the optimal limit of alcohol intake may be higher than in Western populations, despite the high prevalence of people with a facial flushing response. The tradition of ‘liver holidays’ in Japan may partially explain the reasons for the optimal limit of alcohol at high levels.^18^ From a combined analysis of drinking quantity and drinking patterns, we showed that Japanese men who abstain from drinking for 5–6 days with the intake below 150 g/week had a significantly lower risk of cancer and cerebrovascular disease mortality relative to daily drinkers, even though they consume a maximum of 6.5 large bottles of beer in 1 or 2 days. Further, men who abstain from drinking for 1–2 days a week had a lower risk of cancer and cerebrovascular disease mortality than those who drink everyday among light drinkers (<150 g/week), moderate drinkers (150–299 g/week), and heavy drinkers (≥300 g/week), and a lower risk of all-cause mortality in light drinkers. One possible explanation for the relative benefits of liver holidays might be that daily heavy-drinkers are consistently exposed to acetaldehyde compared with liver holiday takers, which may increase their cancer risk. Another possible explanation is the social support: the benefit of light-to-moderate drinking in preventing cardiovascular disease was reported to be enhanced in subjects who receive stronger social support.^36^ In Japan, social drinking is an important social event, especially for middle-aged men: in our study, men in Cohort II who responded to the frequency of social drinking reported that those who take “liver holidays” were more likely to drink on socializing occasions than those who drink almost every day,^18^ suggesting a possible link between liver holidays, social drinking, and social support.

In our study, alcohol intake also showed associations with the risk of heart disease, cerebrovascular disease, and cancer mortality, depending on the amount of drinking. Previous studies reported that regular low-dose drinking is protective against heart disease, mediated via an increase in high-density lipoproteins, lower concentrations of fibrinogen, and inhibition of platelet aggregation.^37^ Light-to-moderate alcohol consumption is also known to exhibit anti-inflammatory effects.^24^ Any of these factors may contribute to minimizing the mortality risk from cardiovascular disease. J-shaped associations with the cancer risk can be explained by the fact that light-to-moderate drinking has been shown to improve immunologic function via increased cell-mediated and humoral immune responses.^38^ A previous study using the same JPHC data showed that light-to-moderate drinking is associated with a lower risk of Non-Hodgkin’s lymphoma^39^ relative to non-drinkers. Moderate alcohol intake is also associated with an improvement in insulin resistance, contributing to a reduced risk of type 2 diabetes mellitus,^40^ which is a risk factor for cancer. However, overdose of alcohol is a common risk factor for multiple morbidities: the International Agency for Research on Cancer reported that alcohol is carcinogenic to humans (Group 1) in different types of cancers,^3^ and acetaldehyde associated with alcohol consumption is a known carcinogen.^41^ Excess intake of alcohol also impedes absorption of dietary folate and its bioavailability,^42^ which contributes to aberrant DNA synthesis and methylation, leading to carcinogenesis.^43^ Our study also indicated that the mortality risk due to all causes, cancer, and cerebrovascular disease in both men and women, and to heart disease in women, may linearly increase when we restricted our analysis to current drinkers.

With regard to respiratory disease mortality, our subgroup analysis by smoking status showed risk attenuation, suggesting residual confounding by smoking. A study in Europe reported a lower risk of respiratory disease death in light-to-moderate drinking men (>0 to ≤60 g/day), but the results were of borderline significance.^7^ Further study is required to investigate the associations between light-to-moderate drinking and respiratory disease mortality.

Further, our study showed a J-shaped association with mortality from injury in both men and women. However, this association might have been due to reverse causality, since people with psychological problems tend to quit drinking or continuously drink extreme amounts. In current drinkers, mortality risk due to injury was found to increase linearly: past studies similarly reported that heavy alcohol intake was associated with increased risk of suicide and violence^44^ and unintentional injuries.^45^

The strengths of the study include prospective and updated analysis of alcohol consumption, in both quantity and frequency of drinking, with a long-term follow-up period and enrollment of more than 100,000 participants, to examine associations between alcohol and mortality risk by gender and by subgroup. Because alcohol use changes over time, updating the information on alcohol intake should improve the accuracy of assessment during the follow-up period.^28^^,^^46^ However, several limitations warrant mention. A certain proportion of drinkers may have been classified as non-drinkers if they rarely consume alcohol. However, such misclassification would only have attenuated the results toward null, and misclassification bias over time is unlikely, since we used measurement of alcohol intake over 10 years. Second, we did not have information on age at onset of alcohol intake or duration of abstaining from alcohol in the past, which restricted our analysis of updated exposure to the follow-up period, instead of lifetime exposure to alcohol. Third, our analyses were constrained by the limited number of heavy-drinking women, which made it difficult to assess mortality risk in women who drink more than 300 g per week. Fourth, validity of alcohol questionnaires was relatively low in women compared with men, but the validity is comparable to previous studies from Japan.^5^^,^^22^

In conclusion, this study suggests J-shaped associations between alcohol intake and the risk of total mortality and three leading causes of death. However, alcohol intake was associated with a linear, positive increase in mortality risk when we restricted our analysis to current drinkers, which highlights the necessity of drinking in moderation coupled with liver holidays.

## APPENDIX A. SUPPLEMENTARY DATA

eTable 1. Adjusted hazard ratios of mortality by alcohol consumption status, excluding past drinkers (Men in Cohort II only)

eTable 2. Adjusted hazard ratios of mortality by alcohol consumption status, excluding past drinkers (Women in Cohort II only)

eTable 3. Adjusted hazard ratios of mortality by smoking status

eTable 4. Analysis of association between alcohol intake and total mortality excluding abstainers during follow-up
