# Supplementary material for: Impact of Alcohol Intake and Drinking Patterns on Mortality From All Causes and Major Causes of Death in a Japanese Population
Source: J Epidemiol. 2018 Mar 5;28(3):140–8. doi: 10.2188/jea.JE20160200 (PMC5821691; doi:10.2188/jea.JE20160200)
Supplement: Supplementary file 1 [file je-28-140-s001.pdf]

eTable 1. Adjusted hazard ratios of mortality by alcohol consumption status, excluding past drinkers (Men in Cohort II only)

	Cumulative Average Intake													
	Non-drinkers	Occasional drinkers		1–149 g/week		150–299 g/week		300–449 g/week		450–599 g/week		600+ g/week		P for non-linear trend
	HR[a]	HR	95% CI	HR	95% CI	HR	95% CI	HR	95% CI	HR	95% CI	HR	95% CI	
**Participants (n=26,023)**	3,636	2,475		6,471		6,427		3,984		1,728		1,302		
All-cause mortality														
Number of cases (n=5,560)	1,091	475		1,232		1,294		827		331		310		
Multivariate HRs[b]	1.00	0.72	(0.64–0.80)	0.76	(0.70–0.82)	0.74	(0.67–0.80)	0.83	(0.75–0.92)	0.82	(0.72–0.94)	1.05	(0.91–1.21)	<0.001
Cancer														
Number of cases (n=2,321)	444	175		540		547		358		138		119		
Multivariate HRs[b]	1.00	0.65	(0.54–0.78)	0.87	(0.77–0.98)	0.80	(0.70–0.92)	0.91	(0.78–1.06)	0.99	(0.81–1.20)	1.03	(0.82–1.29)	<0.001
Heart disease														
Number of cases (n=649)	135	61		127		153		96		40		37		
Multivariate HRs[b]	1.00	0.68	(0.50–0.94)	0.63	(0.49–0.80)	0.63	(0.49–0.81)	0.69	(0.52–0.92)	0.60	(0.41–0.89)	0.90	(0.61–1.33)	<0.001
Cerebrovascular disease														
Number of cases (n=475)	85	35		106		107		81		27		34		
Multivariate HRs[b]	1.00	0.59	(0.39–0.89)	0.74	(0.55–0.98)	0.76	(0.57–1.02)	0.86	(0.61–1.20)	0.87	(0.56–1.35)	1.08	(0.67–1.73)	0.010
Respiratory disease														
Number of cases (n=427)	104	38		101		94		49		18		23		
Multivariate HRs[b]	1.00	0.50	(0.33–0.75)	0.59	(0.45–0.78)	0.52	(0.39–0.70)	0.46	(0.32–0.68)	0.52	(0.31–0.87)	0.86	(0.53–1.42)	<0.001
Injury														
Number of cases (n=421)	65	42		88		96		75		28		27		
Multivariate HRs[b]	1.00	1.05	(0.71–1.55)	0.83	(0.60–1.15)	0.86	(0.61–1.19)	1.07	(0.75–1.54)	0.71	(0.42–1.19)	1.38	(0.85–2.24)	0.075
Other causes														
Number of cases (n=1,268)	258	124		270		297		169		80		70		
Multivariate HRs[b]	1.00	0.66	(0.53–0.83)	0.62	(0.52–0.73)	0.61	(0.51–0.73)	0.62	(0.50–0.77)	0.82	(0.63–1.07)	0.94	(0.70–1.26)	<0.001

[a] Cox proportional hazards models were used. Intake categories are presented by cumulative alcohol consumption updated to 10-year follow-up survey or available time points.

[b] Adjusted for age (years, continuous) and public health center area, smoking status (never, former, <20 cigarettes/day, ≥20 cigarettes/day), BMI (<18.5, 18.5–<25, 25-<30, 30+), flushing response, history of hypertension, history of diabetes, leisure-time sports or physical exercise (<almost daily, almost daily), intake of coffee and green tea (almost never, ≥1 cup/wk, and ≥1 cup/d), energy intake (continuous), intakes of

fruits, vegetables, fish, meat, dairy products (continuous), and job status (employed or unemployed).

eTable 2. Adjusted hazard ratios of mortality by alcohol consumption status, excluding past drinkers (Women in Cohort II only)

	Cumulative Average Intake											
	Non-drinkers	Occasional drinkers		1–149 g/week		150–299 g/week		300–449 g/week		450+ g/week		P for non-linear trend
	HR[a]	HR	95% CI	HR	95% CI	HR	95% CI	HR	95% CI	HR	95% CI	
**Participants (n=30,315)**	19,306	5,385		4,690		663		186		85		
All-cause mortality												
Number of cases (n=3,451)	2,649	422		294		48		18		20		
Multivariate HRs[b]	1.00	0.73	(0.66–0.81)	0.75	(0.66–0.86)	0.86	(0.64–1.16)	1.23	(0.76–1.97)	3.37	(2.13–5.34)	<0.001
Cancer												
Number of cases (n=1,289)	973	163		123		15		8		7		
Multivariate HRs[b]	1.00	0.56	(0.46–0.69)	0.69	(0.56–0.85)	0.78	(0.47–1.27)	1.25	(0.61–2.56)	2.06	(0.90–4.73)	<0.001
Heart disease												
Number of cases (n=490)	385	59		35		4		3		4		
Multivariate HRs[b]	1.00	0.60	(0.43–0.84)	0.62	(0.42–0.91)	0.56	(0.21–1.54)	1.62	(0.50–5.23)	5.13	(1.78–14.78)	<0.001
Cerebrovascular disease												
Number of cases (n=358)	284	40		21		8		1		4		
Multivariate HRs[b]	1.00	0.43	(0.28–0.66)	0.57	(0.36–0.90)	1.14	(0.52–2.50)	0.60	(0.08–4.41)	5.31	(1.78–15.82)	<0.001
Respiratory disease												
Number of cases (n=201)	174	19		5		3		0		0		
Multivariate HRs[b]	1.00	0.44	(0.24–0.79)	0.26	(0.10–0.63)	1.23	(0.38–4.03)	n/a	n/a	n/a	n/a	0.103
Injury												
Number of cases (n=193)	130	26		26		7		3		1		
Multivariate HRs[b]	1.00	0.72	(0.44–1.17)	0.99	(0.62–1.57)	1.50	(0.63–3.58)	3.21	(1.10–9.40)	2.03	(0.27–15.53)	0.055

Other causes												
Number of cases (n=920)	703	115		84		11		3		4		
Multivariate HRs[b]	1.00	0.46	(0.35–0.60)	0.82	(0.64–1.05)	0.78	(0.41–1.49)	1.32	(0.53–3.27)	1.74	(0.54–5.60)	<0.001

[a] Cox proportional hazards models were used. Intake categories are presented by cumulative alcohol consumption updated to 10-year follow-up survey or available time points.

[b] Adjusted for age (years, continuous) and public health center area, smoking status (never, former, <20 cigarettes/day, ≥20 cigarettes/day), BMI (<18.5, 18.5–<25, 25-<30, 30+), flushing response, history of hypertension, history of diabetes, leisure-time sports or physical exercise (<almost daily, almost daily), intake of coffee and green tea (almost never, ≥1 cup/wk, and ≥1 cup/d), energy intake (continuous), intakes of fruits, vegetables, fish, meat, dairy products (continuous), and job status (employed or unemployed).

eTable 3. Adjusted hazard ratios of mortality by smoking status

	Cumulative Average Intake												
	Non-drinkers	Occasional drinkers		1–149 g/week		150–299 g/week		300–449 g/week		450+ g/week		P for non-linear trend	P-interaction
	HR[a]	HR	95% CI	HR	95% CI	HR	95% CI	HR	95% CI	HR	95% CI		
**Men**													
All-cause													
Current smokers													*<0.001*
Number of cases (n=5,912)	936	492		1,115		1,479		1,068		822			
Multivariate HRs[b]	1.00	0.73	(0.65–0.81)	0.80	(0.73–0.87)	0.80	(0.74–0.88)	0.88	(0.80–0.96)	1.06	(0.96–1.18)	<0.001	
Never-smokers													
Number of cases (n=1,631)	370	208		427		292		188		146			
Multivariate HRs[b]	1.00	0.73	(0.61–0.86)	0.75	(0.65–0.87)	0.71	(0.61–0.84)	0.96	(0.79–1.15)	1.11	(0.90–1.37)	<0.001	
Cancer													
Current smokers													*0.066*
Number of cases (n=2,544)	390	186		502		664		468		334			
Multivariate HRs[b]	1.00	0.67	(0.56–0.80)	0.88	(0.78–1.01)	0.87	(0.77–0.99)	0.90	(0.78–1.04)	1.13	(0.96–1.32)	<0.001	
Never-smokers													
Number of cases (n=590)	127	71		169		104		67		52			
Multivariate HRs[b]	1.00	0.72	(0.54–0.96)	0.79	(0.63–1.00)	0.66	(0.50–0.87)	0.87	(0.64–1.19)	1.00	(0.70–1.41)	0.003	
Heart disease													
Current smokers													
Number of cases (n=741)	134	76		140		157		140		94			*0.620*
Multivariate HRs[b]	1.00	0.76	(0.57–1.01)	0.67	(0.53–0.85)	0.55	(0.43–0.7)	0.68	(0.53–0.89)	0.72	(0.54–0.97)	<0.001	
Never-smokers													

	Cumulative Average Intake												
	Non-drinkers	Occasional drinkers		1–149 g/week		150–299 g/week		300–449 g/week		450+ g/week		P for non-linear trend	P-interaction
	HR[a]	HR	95% CI	HR	95% CI	HR	95% CI	HR	95% CI	HR	95% CI		
Number of cases (n=209)	48	21		55		40		23		22			
Multivariate HRs[b]	1.00	0.45	(0.26–0.78)	0.70	(0.48–1.04)	0.69	(0.45–1.07)	0.88	(0.53–1.48)	1.18	(0.69–2.03)	0.004	
Cerebrovascular disease													
Current smokers													
Number of cases (n=523)	73	35		86		147		96		86			*0.139*
Multivariate HRs[b]	1.00	0.58	(0.38–0.87)	0.77	(0.57–1.03)	0.87	(0.65–1.15)	0.89	(0.64–1.22)	1.22	(0.87–1.71)	0.003	
Never-smokers													
Number of cases (n=179)	40	22		43		27		28		19			
Multivariate HRs[b]	1.00	0.64	(0.37–1.10)	0.79	(0.51–1.21)	0.73	(0.45–1.18)	1.32	(0.79–2.20)	1.05	(0.57–1.95)	0.064	
Respiratory disease													
Current smokers													*0.393*
Number of cases (n=367)	69	33		69		87		66		43			
Multivariate HRs[b]	1.00	0.49	(0.31–0.78)	0.63	(0.46–0.87)	0.59	(0.43–0.82)	0.61	(0.42–0.89)	0.79	(0.53–1.18)	0.001	
Never-smokers													
Number of cases (n=109)	33	14		25		21		9		7			
Multivariate HRs[b]	1.00	0.57	(0.30–1.06)	0.57	(0.34–0.96)	0.54	(0.30–0.99)	0.44	(0.18–1.07)	0.76	(0.34–1.67)	0.446	
**Never-smoking women**													
All-cause													
Number of cases (n=4,777)	3,627	669		420		39		11		11			
Multivariate HRs[b]	1.00	0.76	(0.70–0.83)	0.80	(0.72–0.89)	0.82	(0.60–1.13)	0.98	(0.54–1.77)	2.71	(1.49–4.90)	<0.001	

	Cumulative Average Intake												
	Non-drinkers	Occasional drinkers		1–149 g/week		150–299 g/week		300–449 g/week		450+ g/week		P for non-linear trend	P-interaction
	HR[a]	HR	95% CI	HR	95% CI	HR	95% CI	HR	95% CI	HR	95% CI		
Cancer													
Number of cases (n=1,941)	1,423	302		189		16		8		3			
Multivariate HRs[b]	1.00	0.67	(0.58–0.78)	0.68	(0.58–0.81)	0.83	(0.51–1.34)	1.46	(0.69–3.07)	0.92	(0.23–3.67)	<0.001	
Heart disease													
Number of cases (n=610)	470	81		53		5		0		1			
Multivariate HRs[b]	1.00	0.60	(0.45–0.80)	0.81	(0.58–1.11)	1.11	(0.46–2.69)	n/a	n/a	1.99	(0.28–14.24)	0.032	
Cerebrovascular disease													
Number of cases (n=498)	401	61		29		5		0		2			
Multivariate HRs[b]	1.00	0.39	(0.28–0.56)	0.43	(0.28–0.66)	0.88	(0.33–2.36)	n/a	n/a	4.15	(1.02–16.81)	<0.001	
Respiratory disease													
Number of cases (n=248)	217	23		8		0		0		0			
Multivariate HRs[b]	1.00	0.40	(0.23–0.67)	0.36	(0.18–0.73)	n/a	n/a	n/a	n/a	n/a	n/a	0.821	

[a] Cox proportional hazards models were used. Intake categories are presented by cumulative alcohol consumption updated to 10-year follow-up survey or available time points.

[b] Adjusted for age (years, continuous), public health center area, smoking status (never, former, <20 cigarettes/day, ≥20 cigarettes/day), BMI (<18.5, 18.5–<25, 25-<30, 30+), flushing response, history of hypertension, history of diabetes, leisure-time sports or physical exercise (<almost daily, almost daily), intake of coffee and green tea (almost never, ≥1 cup/wk, and ≥1 cup/d), energy intake (continuous), intakes of fruits, vegetables, fish, meat, dairy products (continuous), and job status (employed or unemployed).

eTable 4. Analysis of association between alcohol intake and total mortality excluding abstainers during follow-up

	Participants	Number of cases	Cumulative Average Intake											
			Non-drinkers	Occasional drinkers		1–149 g/week		150–299 g/week		300–449 g/week		450+ g/week		P for non-linear trend
			HR[a]	HR	95% CI	HR	95% CI	HR	95% CI	HR	95% CI	HR	95% CI	
**Men**														
Abstained during follow-up														
Without abstainers[b,c]	30,815	4,429	1.00	0.75	(0.70–0.82)	0.75	(0.71–0.80)	0.75	(0.70–0.80)	0.84	(0.78–0.91)	1.02	(0.94–1.11)	<0.001
**Women**														
Abstained during follow-up														
Without abstainers[b,c]	36,713	2,394	1.00	0.77	(0.71–0.83)	0.83	(0.75–0.91)	1.07	(0.87–1.33)	1.36	(0.95–1.93)	2.14	(1.44–3.19)	<0.001

[a] Cox proportional hazards models were used. Intake categories are presented by cumulative alcohol consumption updated to 10-year follow-up survey or available time points.

[b] Adjusted for age (years, continuous), public health center area, smoking status (never, former, <20 cigarettes/day, ≥20 cigarettes/day), BMI (<18.5, 18.5–<25, 25-<30, 30+), flushing response, history of hypertension, history of diabetes, leisure-time sports or physical exercise (<almost daily, almost daily), intake of coffee and green tea (almost never, ≥1 cup/wk, and ≥1 cup/d), energy intake (continuous), intakes of fruits, vegetables, fish, meat, dairy products (continuous), and job status (employed or unemployed).

[c] Abstainers are those who stopped drinking from 5-y or 10-y follow-up onwards among those who completed all three questionnaires (baseline, 5-y follow-up and 10-y follow-up surveys).
